# The mental health of the child and youth population in Europe: Prevention as paradigm

**DOI:** 10.1192/j.eurpsy.2025.71

**Published:** 2025-08-26

**Authors:** C. M. Díaz-Caneja

**Affiliations:** Institute of Psychiatry and Mental Health, Hospital General Universitario Gregorio Marañón, Madrid, Spain

## Abstract

**Abstract:**

Youth mental health is under significant strain, with long-lasting impacts on European citizens and societies. Alleviating this burden requires a preventive approach. We will review current preventive strategies for youth mental health, including school-based universal, selective, and indicated primary prevention interventions. Additionally, we will discuss novel care systems for youth mental health currently available across Europe and their potential for implementation in various settings.

**Disclosure of Interest:**

None Declared

